# Building towards precision medicine: empowering medical professionals for the next revolution

**DOI:** 10.1186/s12920-016-0183-8

**Published:** 2016-05-10

**Authors:** Scott McGrath, Dario Ghersi

**Affiliations:** School of Interdisciplinary Informatics, University of Nebraska at Omaha, 1110 S 67th Street, Omaha, NE 68182 USA

## Abstract

A new paradigm in disease classification, diagnosis and treatment is rapidly approaching. Known as precision medicine, this new healthcare model incorporates and integrates genetic information, microbiome data, and information on patients’ environment and lifestyle to better identify and classify disease processes, and to provide custom-tailored therapeutic solutions. In spite of its promises, precision medicine faces several challenges that need to be overcome to successfully implement this new healthcare model. In this paper we identify four main areas that require attention: data, tools and systems, regulations, and people. While there are important ongoing efforts for addressing the first three areas, we argue that the human factor needs to be taken into consideration as well. In particular, we discuss several studies that show how primary care physicians and clinicians in general feel underequipped to interpret genetic tests and direct-to-consumer genomic tests. Considering the importance of genetic information for precision medicine applications, this is a pressing issue that needs to be addressed. To increase the number of professionals with the necessary expertise to correctly interpret the genomics profiles of their patients, we propose several strategies that involve medical curriculum reforms, specialist training, and ongoing physician training.

## Background

On January 20, 2015 as part of his State of the Union Address, President Obama announced the Precision Medicine Initiative, a program backed by $215 million aimed at initiating a paradigm shift for modern medicine [1]. The goal of this initiative is to create a more targeted approach to disease prevention and treatment. Instead of using generalized diagnostic protocols and treatment options, the genetic make-up of each individual patient and information about their lifestyle and environment would be directly incorporated into diagnosis and treatment recommendations. The funding would provide $200 million in new spending to the National Institutes of Health (NIH), $10 million for the Food and Drug Administration (FDA), and the remaining $5 million would go to the Office of the National Coordinator for Health Information Technology (ONC) [2].

This multipronged approach is aiming to create the foundation for building precision medicine. It also highlights four principal components that are needed for this endeavor to succeed: (1) data, (2) tools and systems, (3) regulation, and (4) people. The first component addresses the need for robust multifaceted biomedical data sets. These data sets represent the knowledge base from which information supporting precision medicine will be drawn. For example, these data sets will enable quantitative risk estimates for given disorders, help identify new biological markers, and will be instrumental in pinpointing individual variation in responding to drug therapies (pharmacogenomics). The second component involves developing the tools needed to work with these complex data sets. This component also includes building the infrastructure and defining the interoperability rules of the network where the data will be housed and exchanged. The privacy, ethics, and regulatory structure required for that network are included in the third component. The final component is people, the key stakeholders who will be using, building, and maintaining the three previous components.

## Precision medicine

The concepts of precision medicine and its older sibling term, personalized medicine, are not new [3, 4]. The term “personalized medicine” has slightly fallen out of favor due to the possibility of “personalized” being confused with efforts to craft treatments and preventions unique to each individual. The argument against the use of the term “personalized medicine” is that the best physicians have always treated their patients on an individual, personalized level. In contrast, the term “precision medicine” seems to better capture the emphasis on treating and preventing diseases based on genetic, lifestyle-related, and environmental factors [5]. However, the two terms are often still used interchangeably.

How to prepare for this new model of care has been examined and discussed previously [6–8]; however, the Precision Medicine Initiative represents the first formal drive in the United States to integrate precision medicine into healthcare and clinical practice. The U.S. is not the first country to establish a major health initiative aimed at building a massive database of genomic information. Genomics England, a company owned by the United Kingdom Department of Health, was established in July 2013 to sequence the genome of 100,000 British citizens, and has been provided £300 million to accomplish this task [9]. It appears efforts to bring precision medicine to fruition are well on their way.

Another major healthcare change has existed in the United States for several years, and its scope and size diminish the Precision Medicine Initiative to the status of a small start-up in comparison to the likes of Google and Microsoft. This change is the Health Information Technology for Economic and Clinical Health (HITECH) Act, which was included in the American Recovery and Reinvestment Act of 2009. HITECH provided the United States Department of Health and Human Services with $29.5 billion to encourage the adoption of Electronic Health Records (EHRs) and to establish meaningful use for interoperability [10].

The progress of the program has been mixed. From 2009–2013, the percentage of hospitals adopting an EHR system with a basic set of EHR functions (including clinician notes) grew from 12 % to 44 %. However, the adoption of comprehensive.

EHRs only grew from 1.6 % to 16.9 % over the same period [11]. Further, there was a disparity between where adoption was occurring and physician satisfaction levels. Small, nonteaching, and rural hospitals lagged behind their larger peers in adopting EHRs [12], as did smaller private practices and clinics [13]. In a survey conducted by the American Medical Association, satisfaction with EHRs dropped significantly over the past 5 years. In 2009, 61 % of respondents were satisfied or very satisfied with their EHR systems; now only 34 % feel that way [14]. Sites resisting EHR adoption often cite cost, productivity loss, finding an EHR platform that meets the practice’s needs, and training as their principal barriers [15]. The experience with EHR expansion efforts in the US should be used to guide efforts to integrate precision medicine into healthcare. Despite the clinical promise offered by EHRs [16], the implementation process has been challenging. Low satisfaction scores, interoperability problems, and lagging adoption in solo practices and non-primary care practices are a few of the barriers faced by EHRs [17]. Seven years after HITECH was signed into law, efforts continue to overcome these impediments, and without proper planning, precision medicine could face issues similar to those experienced by the EHR expansion.

## Direct-to-consumer genetic tests

In reviewing the three principal components of precision medicine (lifestyle, environment, and genomic information), the most challenging piece may reside with patient genetic information. Using genetic sequencing for prediction and diagnosis has been in place for many years, but in 2007, a new marketplace emerged where customers were able to order their own genetic tests online. Identified as Direct-to-Consumer (DTC), these genetic tests help provide a window into what a precision medical model might look like in the very near future. While the debate about the benefits and ethics of these tests still continues [18–20], they have provided a test bed to examine what works, and what areas need improvement.

There are a variety of DTC tests available, and they can cover a wide range of options, including tracking ancestry and family history, looking at specific traits, or searching for variants with known increased risk of disease. The HapMap Project helped build the foundation for carrying out genome-wide association studies (GWAS) that searched for variants with disease associations. These studies uncovered genetic variations associated with more than 80 common polygenic diseases. These GWASs focused on single-nucleotide polymorphisms (SNP), which are “sites in the genome sequence of 3 billion nucleotide bases where individuals differ by a single base” [21]. With approximately 10 million such sites contained within the human genome, these SNPs can serve as a shortcut to identify allelic variants of identified genes. These shortcuts can then be linked to increases or decreases in odds ratios (OR) for developing various traits or diseases. This is an area where other disciplines can help primary care physicians interpret the results of DTC tests. With the reliance of some DTC tests on Genome-wide Association Studies (GWAS) to provide an estimate of increased or decreased risk for particular conditions based on specific genetic variants, careful interpretation of the odds ratio and the effect size is paramount to properly inform a patient about the actual magnitude of the risk [22]. Knowledge of biostatistics concepts related to GWAS can be a valuable resource for assessing the risks and the effect size associated with a variant.

Currently, DTC tests focus on SNP analysis. However, future DTC tests will continue to grow in their complexity as the traditional monogenic tests give way to more advanced multigenic tests. When precision medicine becomes standard practice, the expectation is that most information will come from genomic tests. A critical factor that will determine whether these tests will be valuable in the clinical domain is their correct interpretation by clinicians, and in particular by primary care physicians. The American College of Medical Genetics and Genomics reference this complexity when they recommended that genetics experts should be available for patient DTC genetic test result consultations [23]. There is evidence that primary care physicians feel ill-equipped to answer questions about DTC tests brought up by their patients. A 2012 study sent out mailers to 2,402 primary care and internal medicine providers in North Carolina, of whom 382 responded. Only 38.7 % (*n =* 148) were aware of DTC tests, and of those who knew about the tests, only 15 % (*n =* 59) felt prepared to answer questions about DTC results [24]. Indeed, even prior to the arrival of DTC tests, most physicians were unable to interpret even simple genetic tests [25]. This is a trend that extends beyond the borders of the United States. In a survey of five European countries (France, Germany, The Netherlands, Sweden and the United Kingdom), primary care physicians (*n =* 3,686, from a sample frame of 139,579) were asked about their ability to carry out basic medical genetic tasks. The findings indicate that 44.2 % classified themselves as not confident, 36.5 % somewhat confident, and only 19.3 % were confident or very confident [26].

There are specialists who are trained to help interpret these genetic test results, including molecular geneticists, clinical geneticists, and genetic counselors. However, the number of them employed in the US healthcare industry and globally is low compared to current and future healthcare needs. As of 2015, there are 3,021 genetic counselors employed in the United States with an additional 135 counselors practicing outside of the US [27]. According to the American Board of Medical Genetics and Genomics (ABMGG), as of July 2014 there are only 1,286 certified clinical geneticists in the world. A total 1,194 of the 1,286 are practicing in the United States, which is only 0.18 % of all physicians in the US [28]. There should be a caveat included with these figures that at the time of data collection international equivalent to the ABMGG or the US based National Society of Genetic Counselors were unknown to us. Thus, we were unable to arrive at sound figures for employment of genetic counselors and clinical geneticist, or their equivalent positions outside the US. Future work may include reaching out to groups like the Australian Society of Genetic Counsellors [29] or other international societies to try and capture an improved global picture.

There are additional certified specialists recognized by the ABMGG; however, the number of clinical geneticist is more than double the closest alternative. Globally, there are 618 certified clinical cytogenetists and 512 certified clinical molecular geneticists. There are 2,904 certificates recognized by the AMBGG across seven categories: clinical biochemical genetics, clinical biochemical molecular genetics, clinical cytogenetics, clinical genetics, clinical molecular genetics, medical biochemical genetics, and PhD medical genetics. Only four of these categories comprise specialists who can directly work with patients, and thus only these are included in our counts here: clinical genetics, clinical biochemical genetics, clinical cytogenetics, and medical biochemical genetics. With these combined numbers of these four categories, there are 2,243 certified professional who are able to work with patients and have expertise in genetics. Going forward, they will be referred to as medical geneticists. Combining this figure with the number of genetic counselors yields only 5,264 specialists. It should be noted that these figures are based off membership and certifications from US-based organizations. These numbers should increase with the inclusion of other international certification boards. However, the relative frequency of medical geneticist and counselors is expected to remain small.

Increased market demand may lead to more genetic counselors and medical geneticists being employed. Even so, if this pattern continues, existing medical professionals may have to intervene to support the demand until training and employment of specialists catches up. Development of new clinical geneticists will continue to grow slowly, since only 50 % of available clinical genetics training slots are currently being filled at US medical schools [30]. Demands for these positions are cited as one reason for the low enrollment figures, but the impact of monetary discrepancy between clinical genetics and other specialties will also be explored later in this paper.

## Proposed actions

There are several factors that could slow or even prevent precision medicine from becoming successfully integrated into healthcare. As highlighted above, there is a shortage of subject matter experts and documented low confidence levels within the ranks of primary care physicians on the topic of genetics. Failing to address these educational and staffing gaps will represent a major stumbling block for precision medicine. Returning to the four components identified as elements for a successful integration of precision medicine (data, tools and systems, regulations, and people), we focus our suggestions for avoiding these pitfalls on the people component.

In order to help streamline the success of the President’s initiative, in addition to any efforts by other nations, changes need to be implemented on two levels: (1) how future physicians are trained, and (2) how current physicians can successfully transition to using precision medicine in the most efficient and effective way for their daily practice. Increased training and employment of specialists in genomics and the technology behind precision medicine will support both future and current physicians through this change. Therefore, we look at three categories where we feel change is needed: medical curriculum reform, specialist training, and ongoing physician training.

### Medical curriculum reform

Based on prior published position statements [31, 32], medical students need additional exposure and training in topics related to genetics and genomics. In order to accomplish this, we suggest the following:

- *Make genetics a core competency*. Like anatomy, genetics should be embedded in each level of training in a medical student’s career (didactic instruction, clinical rotations, and to a smaller degree, specialization).
- *Introduce biomedical and bioinformatics tools to medical students.* Computational methods will be an integral component of precision medicine. Therefore, students should be made aware of the strengths and weakness of approaches like data mining and GWAS, and be able to critically evaluate findings derived from these types of study. Introduction to standard statistical techniques should also be included.
- *Adjust entry requirements for medical schools*. Courses should include exposure to more advanced genetics and genomics for incoming students. Introductory genetics courses may not be sufficient anymore.
- *Offer dedicated training tracks in medical schools*. For example, Baylor College of Medicine offers a Genetics Track Curriculum. Initiatives like this can encourage more physicians to pursue one of the medical genetic specialties.
- *Integrate emerging research into graduate medical education*. Also known as medical residency programs, training should be adjusted to apply emerging research into clinical practice. Precision medicine will create a closer link between research and clinical practice, and students should be prepared to take advantage of this.

### Specialist training

Only pursuing enhanced training for future physicians will not be adequate to meet the increasing demand for trained professionals at all ranks. The number of subject matter experts needs to increase too. This includes genetic counselors, bioinformaticians, and biomedical informatics specialists. In this context, bioinformatics focuses on topics at the cellular and molecular level, whereas biomedical informatics is concerned with the public and patient level of healthcare. Medical geneticists do not fall into the “specialist training” category, since they have attended medical schools and thus serve as more of a direct peer with other physicians. The role of the specialists is complementary to that of the physicians and will also be critical to ensure a successful implementation of the precision medicine model. The American Medical Informatics Association (AMIA) position statements on biomedical informatics training informs the following suggestions [33]:

- *Promote informatics*. Develop strategies to recruit, retain, and educate students in the areas of informatics and genetic counseling.
- *Foundational support*. Encourage foundations to fund bioinformatics and biomedical informatics doctoral programs and research.
- *Encourage additional training in an informatics area*. Current medical professionals should pursue education in nursing informatics, translational informatics, biomedical informatics, or bioinformatics.
- *Establish new fellowships*. In order to encourage additional training by medical professionals, new accredited informatics programs are needed, following the lead of ACGME’s newly accredited clinical informatics fellowship [34].
- *Align the professions with precision medicine aims*. Informatics and genetic counseling curricula and research should be adjusted to be in sync with healthcare policies related to precision medicine.
- *Voyage beyond genomics and DNA*. Precision medicine places a heavy emphasis on genomic data, but in the near future, other assays will become just as important. Future informatics professionals should receive training in RNA-seq, epigenomics, proteomics, metabolomics, and microbiome sequencing. Understanding how to fuse and extract new information from enriched data sets will be a critical skill. Emerging techniques like splicing analysis for splice site mutations [35] and seeking pathway suppression/activation with pathway-specific gene sets [36] will require trained professionals to ensure a proper fit within precision medicine. The work of Chen R. et. al. in developing a personal omics picture by connecting genomic, transcriptomic, proteomic, metabolomic, and autoantibody profiles [37] and the developing “networks medicine” model [38] demonstrates the opportunity in this space.

### On-going physician training

One advantage that precision medicine has over EHRs is that it does not have to change the way every physician operates. In the prior two categories, we emphasized the need for ramping up the number of future specialists. Current medical professionals will be relied upon as new experts matriculate into the system. However, not every physician currently practicing will need to participate, and physician training can be voluntary. To accomplish the goals of this category, we propose the following measures:

- *Introduce new continuing medical education (CME) courses*. CMEs are a standard of training for physicians to ensure medical knowledge remains current. CMEs focused on precision medicine, genomics, and the tools used in these fields should be developed to match the changes implemented in medical curriculum reform.
- *Create precision medicine certification*. Certification status could be reached with given a number of CME courses focused on precision medicine. Organizations like the American Board of Medical Genetics, American Medical Association, or other national-level medical professional organizations could offer these certificates.
- *Proper staffing*. Centers of care should be encouraged to reach a certain percentage of staff that are qualified to discuss precision medicine with patients. This percentage can be reached through genetic counselors, medical geneticists, or physicians who have completed CMEs outlined above.

An important factor to consider besides the adequate training of physicians and specialists is monetary compensation. Key to the success of this revised education model is to ensure that the salaries of medical geneticists and specialists will be competitive enough to attract a sufficient number of qualified individuals to the profession. The average salary for medical genetics is $158,597, which falls well short of other specialties like emergency medicine ($320,419) and pediatrics ($206,961). In comparison to some of the most sought-after specialties like dermatology ($400,898) and orthopedic surgery ($535,688), the discrepancy in pay becomes very apparent. In fact, medical genetics remains one of the lowest paid specialties [39]. The mean annual wage for genetic counselors is $69,540 [40], but that may also not be high enough to attract a sufficient number of professionals to this career.

## Conclusions

The transition to a medical care model where providers are afforded a higher degree of granularity in their treatments and decisions is obviously desirable. Precision medicine offers the potential to tie big data into the equation for medicine. There is tremendous advantage to incorporating big data analytics into healthcare, including R&D acceleration, expanded genomic analysis, and public health insights to name a few [41]. However, big data is not a panacea, and even the best intentions can lead to poor results if efforts in planning are inadequate. In this position paper, we highlighted some of the challenges that EHR implementation has faced as a path that precision medicine may follow if adequate measures are not taken in time. EHRs adoption has been bumpy at best due to multiple factors and poor physician satisfaction ranks highly among those adoption barriers. Finding ways to ease the transition and hire individuals tasked with ensuring clear communication and the capabilities of precision medicine is possible way to avoid the frustrations and disillusionment pitfalls experienced with EHRs adoption.

There is currently a lack of experts ready to usher in this paradigm shift, and current physicians appear to be underequipped to take on the challenge alone. There is still time to correct this, if actions are taken now. We propose ways to address these deficiencies through changes in medical curricula, specialist training, and continuing education for current physicians. The promise of precision medicine is a desirable goal, and we are convinced that through these actions it can arrive sooner and be adequately positioned to have a positive impact on human health at large.

## Abbreviations

ONC: office of the national coordinator for health information technology
HITECH: health information technology for economic and clinical health
EHR: electronic health record
DTC: direct-to-consumer
GWAS: genome-wide association studies
OR: odds ratio
SNP: single-nucleotide polymorphisms
ABMGG: American board of medical genetics and genomics
AMIA: American medical informatics association
ACGME: accreditation council for graduate medical education
CME: continuing medical education
